# How pet food selection attributes influence customer satisfaction and loyalty: evidence from South Korea

**DOI:** 10.3389/fnut.2025.1576518

**Published:** 2025-07-30

**Authors:** Ji-eun Lee

**Affiliations:** Department of Bakery & Cafe, Hotel Culinary Arts & Food Service, Baekseok Culture University, Cheonan-si, Chungcheongnam-do, Republic of Korea

**Keywords:** selection attributes, pet food, customer satisfaction, customer loyalty, South Korea, pet consumer behavior, pet food marketing

## Abstract

**Background:**

This study examined the impact of pet food selection attributes, including price, quality, reliability, and convenience, on customer satisfaction and loyalty in South Korea’s evolving pet food market. The pet industry in South Korea is expanding rapidly, driven by changes in demographics like the rise of single-person households and aging populations. It is essential to understand consumer preferences in pet food attributes.

**Methods:**

A survey of 365 pet owners was conducted, and data were analyzed using path analysis.

**Results:**

The analysis showed that reliability and convenience play a significant role in increasing customer satisfaction and loyalty, while price and quality do not have a significant effect. Consumers value trust in product origins, brand credibility, and practical features like easy-to-store packaging. These results underscore the importance of attributes that simplify daily routines and build trust in pet food products.

**Discussion:**

These findings stress the importance of transparency in production processes and consumer-oriented product designs that meet modern demands for convenience. By prioritizing these attributes, pet food manufacturers can better align their products with changing consumer expectations, improving loyalty and strengthening their competitive position in a dynamic market. This study offers insights into how selection attributes affect customer satisfaction and loyalty, with practical implications for product development and marketing strategies in the rapidly growing pet food sector. However, the study is limited by its sample representativeness and the scope of analyzed attributes, suggesting considerable scope for future research to broaden demographic coverage and explore additional influencing factors.

## 1 Introduction

Pet food is defined here as “food intended for companion animals, excluding other animals and industrial animals (1).” Pet food has evolved to resemble human food as pet owners increasingly consider their pets as family members. Companies respond by developing products and marketing strategies to meet pet owners’ demands (2). As the perception of pets as family members expands and the pet industry grows, pet food has diversified beyond basic feed to include products manufactured with human-grade ingredients and diets tailored for specific pet needs. Given these trends, the South Korean pet food market is expected to experience continuous growth in feed, treatment, and supplemental consumption. The market’s potential is significant, as pet food is a recurring expense that embodies the characteristics of a “food” product (3).

The global pet industry has been steadily expanding, with its market size estimated at $235.32 billion in 2022 and projected to grow to $368.88 billion by 2030 (4). The United States maintains the largest share of the global pet food market, with sales reaching approximately $53.2 billion in 2022 and projected to grow to $62.1 billion by 2028 (5). By contrast, Japan’s pet food market is relatively much smaller, reaching only ¥387.5 billion (approximately $2.9 billion) in 2022, but is showing a steady upward trend driven by increased demand for premium and health-conscious products (6).

Compared to these mature markets, South Korea’s pet food industry is still emerging but beginning to expand rapidly, particularly in the premium segment. By the end of 2022, 25.7% of South Korean households, equating to 5.52 million households, were raising pets – an increase of 2.8% compared to 5.36 million households at the end of 2020. Individuals raising pets have increased to 12.62 million (7). An increase in pet ownership is associated with demographic changes such as smaller family sizes, increased single-person households, longer life expectancy, urbanization, and shifting preferences toward pet ownership (8). Consequently, the pet food market, which provides food products for pets, has garnered attention as the number of pet-owning households grows.

The growing interest in premium pet food products, driven by consumers’ increasing awareness of pet health, has led to a surge in the demand for food products perceived as both high-quality and nutritionally beneficial (9). Compared to pet food markets like the United States that have long matured and are now being shaped by premiumization and health-oriented products (10), South Korea’s pet food market remains relatively young but is rapidly expanding. In the United States, consumers prioritize transparency in labeling and wellness-focused formulations (9), whereas in Japan, regulatory emphasis is placed on consumer safety through detailed labeling standards and ingredient origin disclosure, guided by the Pet Food Safety Act and industry-led fair competition rules (11). These international practices provide valuable benchmarks for interpreting the unique trajectory of South Korea’s emerging pet food industry.

In South Korea, where the premium pet food market is expanding, pet owners actively seek products that align with their values, particularly regarding product quality, safety, and ethical sourcing of ingredients (12). Pet owners favor high-quality and safe options, often choosing higher-priced premium products, including imported brands (3). Furthermore, South Korean pet owners allocated 59.7% of their pet-related expenses to purchasing pet food, with a higher proportion purchasing dry food as a staple. They emphasize their pets’ preferences and price considerations during the purchase process, expressing a preference for South Korean-made products and raw materials but perceiving a limited selection in the market to meet these criteria (12).

With the evolution of the global pet food industry, understanding consumer preferences for product attributes has become increasingly important. Rombach and Dean (13) highlighted the significant impact of natural ingredients, convenience, and health claims on consumer purchasing behaviors in the United States, underscoring the relevance of subjective knowledge about pet food and suggesting that insights into these attributes could also be applicable to other markets. Supporting this, Kwak and Cha (14) found that brand reputation and perceptions of product health significantly influenced consumer attitudes and purchase intentions. Additionally, Park et al. (15) demonstrated that attachment to pets significantly influences the purchasing attributes of pet products, such as price appropriateness, quality, and sales environment. Their findings indicate that deeper attachment to pets enhances the importance placed on these attributes, significantly affecting consumer satisfaction and behavioral intentions.

Given these insights and observed gaps in the literature, the present study aimed to address existing gaps by focusing on the sub-factors of pet food selection attributes, such as price, quality, reliability, and convenience, and examining their impact on customer satisfaction and loyalty. There is a notable scarcity of research that directly investigates the effects of pet food selection attributes on loyalty. In particular, little empirical research has examined how these attributes jointly affect satisfaction and loyalty in the pet food sector. Therefore, this study explored how these attributes influence satisfaction and loyalty, particularly emphasizing their interrelationships. Understanding these dynamics is crucial for pet food manufacturers as they strive to optimize product offerings that meet evolving consumer expectations and foster long-term customer loyalty. By examining these factors, this study aimed to contribute to developing effective marketing strategies and product innovations in South Korea’s growing pet food market.

Given this context, this study addressed the following research questions:

1. How does each of the pet food selection attributes (price, quality, reliability, and convenience) affect customer satisfaction?
2. How do these individual attributes influence customer loyalty?
3. To what extent does customer satisfaction contribute to customer loyalty in the pet food market?

## 2 Literature review

### 2.1 Pet food selection attribute

Selection attributes refer to tangible and intangible factors influencing consumers when purchasing products and engaging in subsequent consumption behaviors. Studies have emphasized that these attributes significantly affect both pre-purchase evaluations and post-purchase satisfaction. Pre-purchase evaluations often highlight product expectations and features (16), while post-purchase evaluations focus on satisfaction and consumption behavior influenced by intrinsic and extrinsic product attributes (17).

These attributes are essential in influencing customer satisfaction and purchase-related attitudes, thus establishing their importance in consumer decision-making. Consequently, various disciplines have studied selection attributes extensively (18). In the context of pet food selection attributes, Kwak and Cha (14) highlighted key factors affecting pet food purchases, such as packaging design, price fairness, brand reputation, and perceived product healthiness. Their study demonstrated that these factors positively affect consumers’ recommendations and attitudes toward pet food, significantly impacting purchase intentions. Similarly, Park and Oh (19) analyzed the impact of pet food selection attributes on repurchase intentions. Their findings highlighted reliability, design, preference, superior quality, and price as critical attributes, with reliability, design, and price exerting significant effects on repurchase intention. Kim (20) further elaborated on the product selection attributes for pet food, identifying reliability, superior quality, accessibility, convenience, informativeness, and safety as the key factors shaping consumer choices.

### 2.2 Customer satisfaction

Customer satisfaction is a critical indicator of how well a product or service satisfies or exceeds customer expectations. This arises from a comparison of the anticipated and actual performances, leading to either satisfaction or dissatisfaction. Satisfaction is achieved when a product’s performance aligns with customer expectations, whereas dissatisfaction occurs when a product falls short (21). Satisfaction is not merely about the immediate performance of a product but also extends to the emotional response elicited by using the product or service. Thus, satisfaction encompasses both tangible and intangible experiences associated with a product, including the emotional fulfillment derived from its use (22). It is essential to foster repeat business and nurture long-term customer relationships, underscoring their broad significance across various studies (23). In the context of online services for pet food subscriptions, research has indicated that elements such as service quality, perceived health benefits, and packaging convenience significantly influence customer satisfaction. These factors contribute to a more complex satisfaction matrix in which both product attributes and service delivery play crucial roles (24).

In addition to the attribute-based framework employed in this study, other theoretical models have been used to examine consumer satisfaction and loyalty. For instance, the theory of planned behavior (TPB) emphasizes behavioral intention driven by attitudes, subjective norms, and perceived control (25). Similarly, the expectation–confirmation theory (ECT) has been applied in consumer research to explain satisfaction because of expectations being met or exceeded post-purchase (22). These models provide theoretical support for examining how consumer expectations and beliefs influence loyalty behaviors in the pet food context.

### 2.3 Customer loyalty

Customer loyalty is defined as a consumer’s favorable attitude and strong attachment toward a specific brand or product, driving them to make repeat purchases over time (26). Loyalty emerges from a deep-rooted commitment to a brand, often leading customers to continue their patronage despite competitive market attempts to sway their preferences (27). It is a pivotal factor in achieving sustainable profitability and positively affects a business’s long-term growth and viability (28). In the pet food market, loyalty is influenced not only by traditional factors such as product quality and customer satisfaction, but also by how well the brand aligns with the consumer’s values and lifestyle choices. Studies show that pet owners’ loyalty is significantly shaped by how well pet food brands meet their specific needs and preferences, which are increasingly centered around health, convenience, and ethical production (29).

## 3 Materials and methods

### 3.1 Research model and hypotheses

**Figure 1:**
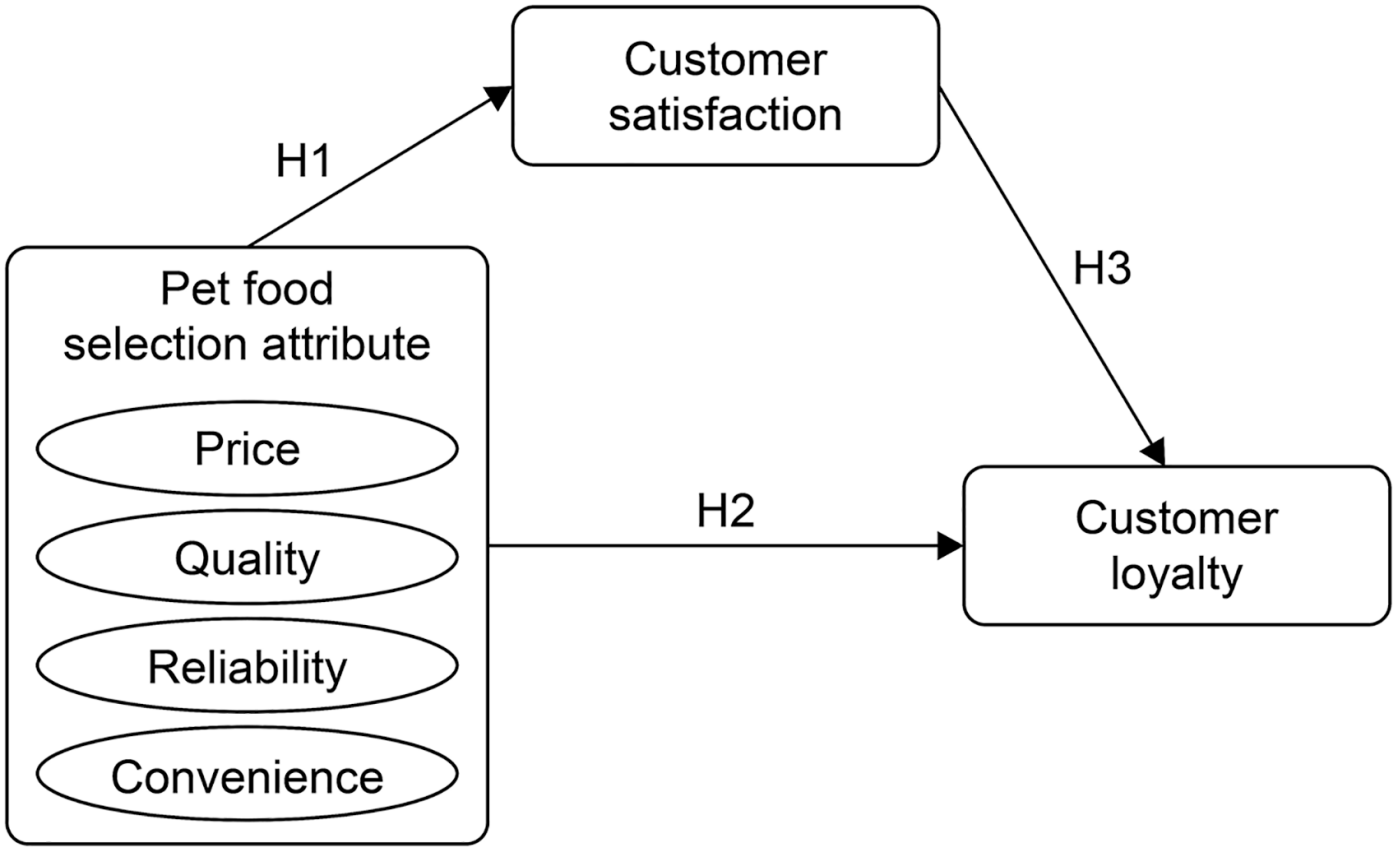
Research model.

This study examined the relationship between pet food selection attributes and their effects on customer satisfaction and loyalty while also exploring the moderating role of lifestyle. Building on the research model proposed by Park and Oh (19), who investigated how pet food selection attributes influence customer satisfaction and repurchase intentions, this study established a framework connecting these attributes to customer satisfaction through a comprehensive literature review. Furthermore, a taste-seeking lifestyle was incorporated as a moderating variable, culminating in the development of the research model depicted in Figure 1.

This study employed structural equation modeling (SEM) using AMOS™ 22.0 to analyze the complex relationships between latent constructs such as selection attributes, customer satisfaction, and loyalty. SEM was selected over traditional regression methods owing to its ability to simultaneously estimate multiple interdependent relationships and account for measurement error in latent variables. AMOS™ was chosen for its strength in visual model specification, its capacity to conduct both confirmatory factor analysis (CFA) and path analysis, and its widespread use in behavioral science research (30, 31).

#### 3.1.1 The relationship between pet food selection attributes and customer satisfaction

In studies on pet food and customer satisfaction, Lee (32) identified quality reliability, price convenience, and preference as the key selection attributes of pet food. This study found that quality and reliability influence all aspects of purchase attitudes, including cognition, emotion, behavior, repurchase intention, and satisfaction. Price convenience affected emotion, behavior, and satisfaction, whereas preference significantly affected cognition, emotion, behavior, and satisfaction. Kim (20) categorized pet food selection attributes as quality, excellence, informativeness, convenience, accessibility, safety, and reliability. Quality excellence, accessibility, convenience, and safety significantly influenced customer satisfaction with pet food products. Based on these studies, we can conclude that pet food selection attributes are important in shaping customer satisfaction. Building on this understanding, this study proposes the following hypotheses:

*H1*: Pet-food selection attributes positively (+) affect customer satisfaction.

*H1-1*: Among the pet food selection attributes, price positively (+) affects customer satisfaction.

*H1-2*: Among the pet food selection attributes, quality positively (+) affects customer satisfaction.

*H1-3*: Among the pet food selection attributes, reliability positively (+) affects customer satisfaction.

*H1-4*: Among pet food selection attributes, convenience has a positive (+) effect on customer satisfaction.

#### 3.1.2 The relationship between pet food selection attributes and customer loyalty

You and Park (33) highlighted that, in terms of behavioral loyalty toward pet food, purchasing attributes play a more significant role than the demographic characteristics of pet owners. Park and Oh (19) found that among pet food selection attributes, reliability, design, and price significantly influenced repurchase intentions, which are closely associated with behavioral loyalty. They emphasized that to encourage customers’ repurchase behavior, it is essential to clearly indicate details such as the product’s manufacturing date and to maintain store cleanliness to enhance customer trust.

These studies suggest a complex interplay between pet food selection attributes, customer loyalty, and repurchase intentions. Furthermore, pet food selection attributes can influence customer loyalty. Based on this insight, the following hypothesis is proposed:

*H2*: Pet food selection attributes have a positive (+) effect on customer loyalty.

*H2-1*: Among the pet food selection attributes, price positively (+) affects customer loyalty.

*H2-2*: Among the pet food selection attributes, quality positively (+) affects customer loyalty.

*H2-3*: Among the pet food selection attributes, reliability positively (+) affects customer loyalty.

*H2-4*: Among the pet food selection attributes, convenience positively (+) affects customer loyalty.

#### 3.1.3 The relationship between customer satisfaction and loyalty

Customer satisfaction is a key driver of long-term customer behavior, significantly influencing actions such as revisit intention and positive word-of-mouth. Promoting customer satisfaction is essential for cultivating loyal and repeat customers (34–36). Park and Oh (19) highlighted customer satisfaction as a mediating factor connecting reliability, design, quality, and repurchase intentions in the context of pet food, emphasizing its importance in enhancing loyalty. Similarly, Park et al. (15) demonstrated that satisfaction with pet-related products plays a crucial role in shaping behavioral intentions as satisfied customers are more inclined to repurchase, continue using a product, and recommend it to others.

Based on these studies, customer satisfaction influences loyalty. Accordingly, the following hypothesis regarding satisfaction and loyalty is proposed in this study:

*H3*: Customer satisfaction with pet food positively (+) affects customer loyalty.

### 3.2 Analytical approach

The survey data were analyzed using AMOS™ 22.0 and SPSS^®^ 23.0. First, a frequency analysis examined the respondents’ demographic characteristics and pet care traits. An exploratory factor analysis (EFA) was applied to validate the measurement tools by identifying the underlying dimensions of pet food selection attributes, customer satisfaction, and loyalty. This process helped ensure that the measurement items reflected the constructs they intended to measure. An internal consistency reliability (CR) analysis was performed to evaluate the reliability of the measurement items, with a focus on Cronbach’s alpha. Loyalty, as reflected in customers’ inclination to repurchase, continued use of a product, and recommendation of the product to others, was used to identify the overall trends and assess the normality of the key variables, ensuring that the data met the assumptions necessary for further analysis. Pearson’s correlation analysis was conducted to explore the relationships among the variables and identify potential linear associations among pet food selection attributes, customer satisfaction, and loyalty.

Confirmatory factor analysis (CFA) was performed to verify the validity of the measurement model, following guidelines suggested by Hair (30) and Kline (31). This process aimed to assess how well the hypothesized model fits the data and whether the measurement items adequately represented the constructs using fit indices such as CFI, RMSEA, and RMR.

AMOS™ was also employed to conduct a path analysis, as path analysis allows the researcher to examine direct effects among observed variables while estimating the strength and significance of hypothesized relationships (31, 37).

Additionally, emphasizing that only a path analysis was used, AMOS™ was used for the path analysis to investigate the causal links between pet food selection attributes, customer satisfaction, and loyalty. Through the path analysis, this study aimed to identify how pet food selection attributes such as price, quality, reliability, and convenience impact customer satisfaction and loyalty. The steps in the path analysis included specifying the measurement model, assessing model fit using various fit indices, and testing the hypotheses regarding the relationships between the variables. This allowed a more detailed exploration of the effects of pet food selection attributes on customer satisfaction and loyalty.

### 3.3 Data collection

This study examined how pet food selection attributes influence customer satisfaction and loyalty. A preliminary survey was conducted from September 27 to October 2, 2024, targeting adults aged 20 and older with pet food purchasing experience. Fifty questionnaires were distributed by convenience sampling. Based on the preliminary survey results, a pretest was conducted to refine and revise certain items for clarity and relevance. The main survey was conducted from October 10 to October 19 using a convenience sampling method. The survey targeted pet owners with prior experience in purchasing pet food and was conducted in a self-administered format, following an explanation of the study’s purpose and objectives. Before participation, all respondents provided informed consent, and their responses were anonymized to ensure confidentiality. This study did not require approval from an institutional review board (IRB) because it involved a non-clinical, anonymous, and voluntary survey of adults that did not require collecting personally identifiable or sensitive information. The research was conducted in accordance with ethical principles for social science research, and all procedures followed informed consent protocols. Survey responses were collected from participants across various regions of South Korea, including metropolitan and non-metropolitan areas. This nationwide sampling approach was adopted to improve the generalizability of the findings. Of the 380 questionnaires distributed, 370 were collected for a response rate of 97.4%. After excluding five questionnaires due to duplication or incomplete responses, 365 valid questionnaires were finalized for the analysis.

The survey was conducted using a mixed-mode approach that included both online and offline formats to accommodate diverse respondent accessibility. The online questionnaire was distributed via Google Forms, while offline paper-based questionnaires were administered in person, especially targeting older adults less familiar with digital tools. All responses were collected using a self-administered method, and participation was entirely voluntary following informed consent procedures. This mixed-mode survey approach was adopted to enhance response accessibility and increase response rates, aligning with previous research that demonstrates the effectiveness of combining web-based and paper-based questionnaires (38).

Selection attributes refer to the characteristics used to compare various alternatives while acquiring and using economic goods or services and making decisions beforehand. Consumers consider these attributes important when purchasing products and services (20, 39). In this study, pet food selection attributes were identified as important factors in purchasing pet food products. The measurement items for pet food selection attributes were reconstructed based on studies by Lee (32), Park and Oh (19), Kim (20), and Park et al. (15) to fit the objectives of this research. Pet food selection attributes included price, quality, reliability, and convenience, evaluated on a 5-point Likert scale (1 = not at all important, 5 = very important). Customer satisfaction refers to the overall psychological state arising from a combination of emotions due to discrepancies between actual experiences, expectations, and pre-purchase emotions (22). In this study, customer satisfaction refers to the level of contentment that customers feel regarding both the product and purchasing process. The scale for customer satisfaction was adapted from the satisfaction scale used by Lee (32) to align with the objectives and target of this study. This was measured using a 5-point Likert scale (1 = strongly disagree to 5 = strongly agree).

Customer loyalty can be measured by repurchasing preferred products and services, recommending them, or demonstrating word-of-mouth intention (40). In this study, customer loyalty was defined as the intention to repurchase, recommend, or engage in word-of-mouth after using a product. Lee (18) and Park and Oh (19) reconstructed a scale for customer loyalty based on the scales used in studies on customer loyalty. This was measured using a 5-point Likert scale (1 = strongly disagree to 5 = strongly agree).

The questionnaire for this study was developed by revising and supplementing measurement scales derived from previous research, as shown in Table 1. The compositions and sources were as follows:

**Table 1:** Composition and sources of the questionnaire.

Factor		Source	Number of items	Scale
Pet care traits	Type of pet, number of pets, duration of ownership, average monthly spending on pet food, frequency of pet food purchases	Park and Um (56)	5	Nominal scale
Pet food selection attributes	Price, quality, reliability, convenience	Lee (32), Park and Oh (19), Kim (20), Park et al. (15)	12	Likert 5-point scale
Customer satisfaction	Customer satisfaction	Lee (32)	4	
Customer loyalty	Customer loyalty	Lee (18), Park and Oh (19)	3	
Demographic characteristics	Sex, age, educational background, occupation, income, marital status	Lee (18)	7	Nominal scale
Total		31 Items		

Descriptive statistics were also used to verify whether the measured variables satisfied multivariate normality. The mean, standard deviation, and minimum and maximum values for each variable are presented in Table 2. Examination of the skewness and kurtosis of the measured variables showed that the skewness ranged from −1.512 to −0.426, which is within the threshold of 3. The kurtosis ranged from −0.502 to 2.399, remaining below the threshold of 7, indicating that the criteria for multivariate normality were satisfied (41).

**Table 2:** Descriptive statistics results.

Variables	Minimum	Maximum	Mean	Standard Deviation	Variance	Skewness	Kurtosis
Price	2.00	5.00	4.063	0.656	0.430	−0.705	0.258
Quality	2.00	5.00	4.319	0.509	0.259	−0.910	1.229
Reliability	2.33	5.00	4.170	0.549	0.301	−0.759	0.504
Convenience	1.67	5.00	4.090	0.616	0.380	−0.910	0.759
Customer Satisfaction	1.67	5.00	4.129	0.566	0.320	−1.104	1.069
Customer Loyalty	3.00	5.00	4.207	0.571	0.326	−0.426	−0.502

The minimum values in Table 2 represent averaged scores across multi-item constructs, resulting in non-integer values despite the use of a 5-point Likert scale.

## 4 Results

### 4.1 Demographic characteristics and pet care traits of respondents

The demographic information and pet ownership characteristics of the participants are presented in Table 3. Of the 365 participants, 114 (31.2%) were male and 251 (68.8%) female, indicating a predominance of female respondents. Regarding age, 171 respondents (46.8%) were in their 30s, 122 (33.4%) were in their 20s, 45 (12.3%) were in their 40s, and 27 (7.4%) were aged 50 years or older. Regarding education level, most respondents were college graduates (260, 71.2%), followed by high school graduates (87, 23.8%), graduates with graduate-level education or higher (16, 4.4%), and those with less than a high school education (2, 0.5%). Occupationally, 229 respondents (62.7%) were office workers or public servants, followed by students (36, 9.9%), professionals (35, 9.6%) business owners (self-employed) (31, 8.5%), homemakers (21, 5.8%), and others (13, 3.6%) others. Monthly income levels showed that 157 respondents (43.0%) earned 3.01–4.00 million KRW (approx. 2,315–3,077 USD), 85 (23.3%) earned 2.01–3.00 million KRW (approx. 1,546–2,308 USD), 56 respondents (15.3%) earned 4.01–5.00 million KRW (approx. 3,085–3,846 USD), while 11 respondents (3.0%) earned less than 2.00 million KRW (approx. 1,538 USD). A household size analysis revealed that 148 respondents (40.5%) lived in single-person households, followed by three (76, 20.8%), two (73, 20.0%), and four or more (68, 18.6%) persons per household. Regarding marital status, 259 respondents (71.0%) were single, and 106 (29.0%) were married.

**Table 3:** General characteristics of sample (*N* = 365).

Demographic characteristics		*N*	%
Gender	Men	114	31.2
	Women	251	68.8
Age	20s	122	33.4
	30s	171	46.8
	40s	45	12.3
	50s or older	27	7.4
Education	Below high school	2	0.5
	High school graduate	87	23.8
	College/University graduate	260	71.2
	Postgraduate degree or higher	16	4.4
Occupation	Student	36	9.9
	Office worker/civil servant	229	62.7
	Professionals	35	9.6
	Business owner (self-employed)	31	8.5
	Housewives	21	5.8
	Others	13	3.6
Monthly income	2 million KRW (approx. 1,538 USD) or less	11	3.0
	2.01 million–3 million (approx. 1,546–2,308 USD)	85	23.3
	3.01 million–4 million (approx. 2,315–3,077 USD)	157	43.0
	4.01 million–5 million (approx. 3,085–3,846 USD)	56	15.3
	5.01 million KRW (approx. 3,854 USD) or more	56	15.3
Family number	1	148	40.5
	2	73	20.0
	3	76	20.8
	More than 4	68	18.6
Marital status	Single	259	71.0
	Married	106	29.0

Pet care traits		*N*	%
Types of pets owned	Dog	246	67.4
	Cat	91	24.9
	Both dog and cat	17	4.7
	Other animals	11	3.0
Number of pets owned	1 pet	250	68.5
	2 pets	98	26.8
	3–4 pets	12	3.3
	5 or more pets	5	1.4
Duration of pet ownership	less than 1 year	23	6.3
	1 to less than 5 years	190	52.1
	5 to less than 10 years	113	31.0
	10 years or more	39	10.7
Average monthly expenditure on pet food	Less than 50,000 KRW (approx. 38 USD)	44	12.1
	50,000 KRW to less than 100,000 KRW (approx. 38–77 USD)	195	53.4
	100,000 KRW to less than 200,000 KRW (approx. 77–154 USD)	86	23.6
	200,000 KRW or more (approx. 154 USD)	40	11.0
Frequency of pet food purchases (Per purchase)	Less than a week	43	11.8
	2–3 weeks	184	50.4
	One month	132	36.2
	Other	6	1.6

As regards pet ownership, 246 respondents (67.4%) owned dogs, 91 (24.9%) owned cats, 17 (4.7%) owned both dogs and cats, and 11 (3.0%) owned other pet types. Regarding the number of pets owned, 250 respondents (68.5%) owned one pet, 98 (26.8%) owned two pets, 12 (3.3%) owned three to four pets, and 5 (1.4%) owned five or more pets. The duration of pet ownership varied as follows: 190 respondents (52.1%) reported owning pets for 1–5 years, 113 (31.0%) for 5–10 years, 39 (10.7%) for more than 10 years, and 23 (6.3%) for less than 1 year. The most common monthly expenditure on pet food was 50,000–100,000 KRW (approx. 38–77 USD) (195, 53.4%), followed by 100,000–200,000 KRW (approx. 77–154 USD) (86, 23.6%), less than 50,000 KRW (approx. 38 USD) (44, 12.1%), and over 200,000 KRW (approx. 154 USD) (40, 11.0%). With reference to frequency of pet food purchases, most respondents (184, 50.4%) purchased pet food once every 2–3 weeks, followed by those who purchased it once a month (132, 36.2%), once a week or more (43, 11.8%), and others (6, 1.6%).

### 4.2 Validity and reliability analysis

The findings of the exploratory factor and reliability analyses of the scales are shown in Table 4. To assess the construct validity of the scales, exploratory factor analysis (EFA) was conducted using principal component analysis with varimax rotation. Following widely accepted criteria, factor loadings of 0.4 or higher were considered significant (42). While some scholars recommend higher cutoffs for smaller samples, (57) notes that for sample sizes exceeding 100, a threshold of 0.4 is generally acceptable. Items with factor loadings below the minimum threshold of 0.4 were sequentially removed, and a varimax rotation factor analysis was repeatedly performed. Common factors with eigenvalues of 1.0 or higher were extracted, resulting in the identification of seven final factors. One item each for price (PR3), customer satisfaction (CS4), and customer loyalty (CL3) were removed.

**Table 4:** Validity and reliability analysis results of selection attributes, satisfaction, and loyalty for pet food.

Factors	Item number	Descriptions	Factor loading	Eigen value	Explanation ratio (%)	Cronbach’s α
Convenience	CO1	Convenience of packaging	0.748	1.995	12.469	0.732
	CO2	Convenience of serving	0.788			
	CO3	Convenience of product storage (for continuous feeding)	0.659			
Quality	QU1	Nutritional components	0.804	1.757	10.983	0.603
	QU2	Safety	0.694			
	QU3	Expiration date or manufacturing date	0.624			
Reliability	RE1	Country of origin (manufacturing country)	0.737	1.709	10.680	0.609
	RE2	Overall trustworthiness of manufacturing processes and regulatory aspects	0.587			
	RE3	Manufacturer brand	0.756			
Customer satisfaction	CS1	I am satisfied with the price of the pet food	0.556	1.698	10.610	0.613
	CS2	I am satisfied with the quality of the pet food	0.715			
	CS3	I am satisfied with the reliability of the pet food	0.767			
Customer loyalty	CL1	I will repurchase the product I have chosen	0.836	1.614	10.090	0.612
	CL2	I will tell others about the benefits of this pet food	0.661			
Price	PR1	Reasonable price	0.782	1.453	9.083	0.609
	PR2	Lower price compared to competitors	0.803			

KMO = 0.793, Bartlett’s sphericity test = 1,321.200, Sig. = 0.000.

The extracted factors were price, quality, reliability, convenience, customer satisfaction, and customer loyalty. The total variance was 63.915%, with a Kaiser–Meyer–Olkin (KMO) value of 0.793 and Bartlett’s test of sphericity yielding a value of 1,321.200 (*p* < 0.001), confirming the appropriateness of the factor analysis. These values meet the commonly recommended thresholds of KMO > 0.6 and *p* < 0.05 for Bartlett’s test (43), supporting the adequacy of the sample for factor analysis.

The results of the reliability analysis for each factor showed the following Cronbach’s alpha values: price (0.609), quality (0.603), reliability (0.609), convenience (0.732), customer satisfaction (0.613), and customer loyalty (0.612). All values were above the 0.6 threshold, indicating that the internal consistency of each factor was valid (44). These reliability coefficients support the internal consistency of the scales and are consistent with recommendations from Nunnally and Bernstein (45) and Tavakol and Dennick (46).

### 4.3 Measurement model validation and structural relationships

To validate the measurement model, both convergent and discriminant validities were assessed after confirmatory factor analysis (CFA).

According to commonly accepted criteria, incremental fit index (IFI) and comparative fit index (CFI) values above 0.9 are considered indicative of good model fit (47, 48), while root mean square residual (RMR) values below 0.08 indicate good fit (37). Root mean square error of approximation (RMSEA) values below 0.08 are considered acceptable (49). Goodness of fit (GFI) values above 0.9 are generally deemed acceptable (50), and adjusted goodness of fit (AGFI) values above 0.9 are also considered adequate (51).

Based on these standards, The CFA results indicated the following model fit indices: *χ*^2^ = 209.561, df = 89, *p* = 0.000, CMIN/df = 2.355, RMR = 0.024, GFI = 0.936, AGFI = 0.902, normed fit index (NFI) = 0.844, IFI = 0.904, CFI = 0.902, and RMSEA = 0.061. These results fell within acceptable thresholds, confirming that the conditions necessary for applying the path analysis model were met.

Table 5 presents the parameter estimates and statistical significance of the convergent validity model. Convergent validity the composite reliability (CR) values exceeded 0.7 are generally considered acceptable for confirming internal consistency and convergent validity (52). In this study, the CR values ranged from 0.725 to 0.827, all exceeding 0.7, thereby confirming that convergent validity was established for all constructs.

**Table 5:** Path coefficients, AVE, and CR of the measurement model.

Latent variable	→	Measured variable	Unstandardized coefficient (B)	Standardized coefficient (β)	CR	SE	AVE	CR
Price	→	PR1	1.136	0.718	6.458^***^	0.176	0.571	0.725
		PR2	1.000	0.609				
Quality	→	QU1	1.285	0.191	6.720^***^	0.191 0.177	0.542	0.778
		QU2	1.162	0.177	6.562^***^			
		QU3	1.000					
Reliability	→	RE1	1.349	0.669	7.313^***^	0.184	0.501	0.749
		RE2	1.038	0.558	6.833^***^	0.152		
		RE3	1.000	0.534				
Convenience	→	CO1	1.241	0.722	10.123^***^	0.123	0.616	0.827
		CO2	1.180	0.707	10.023^***^	0.118		
		CO3	1.000	0.648				
Customer satisfaction	→	CS1	0.931	0.534	7.035^***^	0.132	0.490	0.742
		CS2	0.974	0.629	7.615^***^	0.128		
		CS3	1.000	0.618				
Customer loyalty	→	CL1	0.830	0.627	7.597^***^	0.109	0.641	0.781
		CL2	1.000	0.705				

CR, composite reliability; AVE, average variance extracted; SE, standard error; ^***^p < 0.001; *χ*^2^ = 209.561(df = 89), *p* = 0.000, CMIN/df = 2.355, RMR = 0.024, GFI = 0.936, AGFI = 0.902, NFI = 0.844, IFI = 0.904, CFI = 0.902, RMSEA = 0.061.

Discriminant validity was assessed based on the average variance extracted (AVE). An AVE value above 0.5 is typically considered indicative of adequate discriminant validity (18). The AVE value for price, quality, reliability, convenience, and customer loyalty ranged from 0.501 to 0.641, satisfying the threshold. However, the AVE value for customer satisfaction was slightly below 0.5, indicating that discriminant validity was partially established in the measurement model.

### 4.4 Analysis of discriminant validity and variable correlations

Correlation analysis among variables revealed positive correlations (*p* < 0.001) between pet food selection attributes—price, quality, reliability, and convenience—and customer satisfaction, customer loyalty, and taste-seeking lifestyles. These results align with the hypotheses proposed in this study.

Discriminant validity was assessed using both the Fornell–Larcker criterion and the heterotrait–monotrait ratio (HTMT). According to the Fornell–Larcker criterion, the square root of the AVE for each latent variable (as shown along the diagonal in Table 6) exceeded the correlation coefficients between constructs, indicating acceptable levels of discriminant validity (53).

**Table 6:** Discriminant validity of the measurement model.

	Variables	1	2	3	4	5	6
1	Price	(0.753)	0.168	0.182	0.258	0.151	0.192
2	Quality	0.265^***^	(0.736)	0.203	0.222	0.124	0.164
3	Reliability	0.288^***^	0.362^***^	(0.708)	0.253	0.173	0.200
4	Convenience	0.376^***^	0.367^***^	0.420^***^	(0.785)	0.223	0.248
5	Customer Satisfaction	0.240^***^	0.217^***^	0.309^***^	0.368^***^	(0.700)	0.265
6	Customer Loyalty	0.268^***^	0.258^***^	0.317^***^	0.364^***^	0.415^***^	(0.802)

The square root of the AVE value is shown along the diagonal, and the correlation coefficients between the latent variables are displayed below the diagonal. The upper triangle displays the HTMT values. ^***^p < 0.001.

To assess discriminant validity, the HTMT values were examined. As shown in Table 6, all HTMT values among the constructs were below the threshold of 0.85, ranging from 0.124 to 0.265, thereby confirming acceptable discriminant validity (54).

These analyses confirm that the constructs are conceptually distinct and methodologically robust, satisfying the requirements for discriminant validity.

### 4.5 Hypothesis testing

Path analysis was conducted to examine the impact and relationships between pet food selection attributes, customer satisfaction, and loyalty. Table 7 summarizes the results of the hypothesis tests. The analysis demonstrated that the research model exhibited a perfect fit, and fit indices were omitted.

**Table 7:** Results of hypothesis testing and path coefficients.

Hypothesis	Path			Specific path			Non-standardized coefficient		Standardized coefficient	*p*	CR (t)
							*B*	SE	*β*		
H1	Pet food selection attributes	→	Customer satisfaction	Price	→	Customer satisfaction	0.075	0.045	0.087	0.097NS	1.662
				Quality	→		0.047	0.059	0.042	0.425NS	0.798
				Reliability	→		0.168	0.056	0.163	0.003**	2.982
				Convenience	→		0.231	0.052	0.251	0.000***	4.468
H2	Pet food selection attributes	→	Customer loyalty	Price	→	Customer loyalty	0.079	0.044	0.090	0.071NS	1.806
				Quality	→		0.086	0.057	0.077	0.130NS	1.515
				Reliability	→		0.117	0.055	0.112	0.033*	2.134
				Convenience	→		0.138	0.051	0.149	0.007**	2.715
H3	Customer satisfaction	→	Customer loyalty	Customer satisfaction	→		0.290	0.050	0.287	0.000***	5.766

SE, standard error; CR, composite reliability; NS, not significant; *p < 0.05, **p < 0.01, ***p < 0.001.

For Hypothesis H1, “Pet food selection attributes will have a positive (+) effect on customer satisfaction,” the results showed that Reliability and Convenience had significant positive effects on customer satisfaction, whereas Price and Quality did not demonstrate significant relationships. Specifically, for H1-1 (“Price as a pet food selection attribute will have a positive (+) effect on customer satisfaction”), the *β* value was 0.087, the CR (t) value was 1.662, and the *p*-value was not significant. For H1-2 (“Quality as a pet food selection attribute will have a positive (+) effect on customer satisfaction”), the *β* value was 0.042, the CR (t) value was 0.798, and the *p*-value was also not significant. However, for H1-3 (“Reliability as a pet food selection attribute will have a positive (+) effect on customer satisfaction”), the *β* value was 0.163, the CR (t) value was 2.982, and the *p*-value was below 0.01, indicating a significant positive influence. Similarly, for H1-4 [“Convenience as a pet food selection attribute will have a positive (+) effect on customer satisfaction”], the *β* value was 0.251, the CR (t) value was 4.468, and the *p*-value was below 0.001, demonstrating a significant positive influence.

These findings suggest that reliability and convenience are the most influential factors that affect customer satisfaction, with convenience being the most significant selection attribute. This is consistent with previous studies, such as those by Kim and Lee (55), who reported that consumers focusing on convenience showed the highest satisfaction. Similarly, Kim (20) found that convenience significantly affected customer satisfaction.

For Hypothesis H2, “Pet food selection attributes will have a positive (+) effect on customer loyalty,” the results revealed that reliability and convenience significantly influenced customer loyalty, while price and quality did not show any significant impact (*p* > 0.05). Specifically, for H2-3 (“Reliability as a pet food selection attribute will have a positive (+) effect on customer loyalty”), the *β* value was 0.112, the CR (t) value was 2.134, and the *p*-value was below 0.05, demonstrating a significant positive influence. Likewise, for H2-4 (“Convenience as a pet food selection attribute will have a positive (+) effect on customer loyalty”), the *β* value was 0.149, the CR (t) value was 2.715, and the *p*-value was below 0.01, showing a significant positive influence. Park and Oh (19) found that price and reliability, among pet food selection attributes, influence repurchase intention, presenting findings that are partially consistent with those of this study.

For Hypothesis H3, “Customer satisfaction with pet food will have a positive (+) effect on customer loyalty,” the *β* value was 0.287, the CR (t) value was 5.766, and the *p*-value was below 0.001, reflecting a significant positive impact, thus supporting the hypothesis.

These results confirm that convenience and reliability are crucial drivers of customer satisfaction with and loyalty to the pet food market. The positive relationship between customer satisfaction and loyalty is also consistent with the findings of Lee (18), who found a strong correlation between customer satisfaction and loyalty in similar contexts.

**Figure 2:**
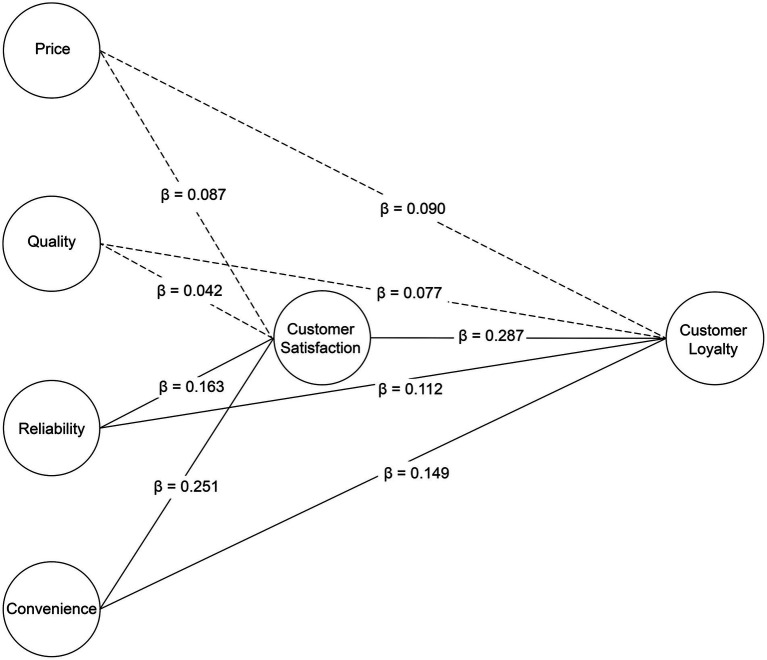
Visual path diagram.

To enhance interpretability, a visual path diagram (Figure 2) has been added to illustrate the standardized coefficients among the variables, emphasizing the central role of reliability and convenience in influencing satisfaction and loyalty. This visual summary clarifies that price and quality attributes did not have significant effects, whereas reliability and convenience were the only selection attributes that significantly influenced both satisfaction and loyalty. Notably, these two attributes exerted a direct influence on customer loyalty that was stronger than their indirect influence via satisfaction, highlighting their critical and independent roles in shaping consumer behavior.

These results support prior research indicating that consumer trust and ease of use are powerful drivers in food-related purchases (9, 19, 29). For instance, Schleicher et al. (29) emphasized that perceived product reliability enhances brand commitment, while White (9) showed that convenience features and product transparency drive loyalty in premium pet food categories. Park and Oh (19) similarly identified design and reliability as determinants of repurchase behavior. The current findings are consistent with these studies, reinforcing the idea that practical value and trustworthiness are more influential than price or satisfaction alone in the context of pet food purchasing.

## 5 Discussion

### 5.1 Relevance and key findings

As the number of pet-owning households continues to rise globally, the pet industry is expanding rapidly, highlighting the growing importance of the “petconomy” and the increasing demand in the pet food market. In South Korea, despite demographic trends such as the rise of single-person households, an increase in unmarried individuals, and an aging population, the number of pet-owning households is consistently increasing. These trends suggest that the pet food sector will likely play a significant role in South Korea’s economy and continue growing. This study is particularly relevant as it provides timely insights into consumer behavior in the South Korean pet food market, which is undergoing a rapid premiumization process. The results can guide product development and marketing strategies tailored to evolving consumer expectations, making the study highly applicable to both domestic and emerging pet food markets.

This study aimed to contribute to the long-term development of South Korea’s pet food industry by analyzing the factors influencing consumer selection of pet food and the impact of these factors on customer satisfaction and loyalty. The findings are as follows: Four selection attributes—price, quality, reliability, and convenience—were validated using confirmatory factor and reliability analyses. The path analysis evaluated the impact of pet food selection attributes on customer satisfaction and loyalty. The results revealed that all six factors (price, quality, reliability, convenience, customer satisfaction, and customer loyalty) had reliability values exceeding 0.6, with the CR and AVE values partially supporting the validity of the measurement model, thus ensuring that the data were significant.

### 5.2 Comparison with prior research

The path analysis results for H1, “Pet food selection attributes will have a positive (+) effect on customer satisfaction,” indicated that reliability and convenience significantly impacted customer satisfaction. By contrast, price and quality did not significantly influence customer satisfaction. Similarly, for H2, “Pet food selection attributes have a positive (+) effect on customer loyalty,” reliability and convenience have significant positive effects on customer loyalty, while price and quality do not. Finally, for H3, “Customer satisfaction will have a positive (+) effect on customer loyalty,” the results show that customer satisfaction significantly impacts customer loyalty, indicating a positive correlation.

While prior studies have emphasized the role of price in shaping consumer behavior, the findings of this study offer a contrasting perspective. For instance, Park et al. (15) found that price positively influenced satisfaction in pet product purchases, and Kwak and Cha (14) reported that price fairness significantly affected consumers’ intention to recommend pet food products. However, in this study, price did not significantly impact either satisfaction or loyalty, suggesting that in the context of Korea’s premiumized pet food market, other attributes such as reliability and convenience may override price sensitivity. This divergence highlights the evolving criteria consumers use when evaluating pet food, especially in rapidly developing markets.

### 5.3 Managerial implications for the pet food industry

The findings confirm that factors such as product origin, brand, manufacturing process, and regulatory compliance significantly affect customer satisfaction with pet food, emphasizing the importance of building consumer trust. These results are consistent with those of previous studies that emphasize the significance of reliability and transparency in the manufacturing process. Consumers value clear information regarding the origin and production processes of pet food products, which strongly influence their purchasing decisions. Consequently, pet food manufacturers should prioritize transparency, provide credible certifications, and disclose quality control data to build consumer trust.

In addition, this study revealed that convenience factors such as packaging usability, ease of feeding, and storage convenience significantly impact customer satisfaction. This finding aligns with Kim (20), who emphasized that convenience, particularly regarding packaging and feeding ease, had a significant positive effect on customer satisfaction in the context of pet food selection. His study also highlighted the practical importance of usability in packaging as a key determinant of satisfaction, consistent with the current findings.

Consumers prioritize not only the health and satisfaction of their pets but also their own convenience when selecting pet food. Therefore, manufacturers should focus on incorporating convenience into product design and development. This could include packaging that facilitates feeding and maintains product freshness with convenient storage options for non-disposable products. By focusing on convenience, manufacturers can improve customer satisfaction and loyalty while differentiating their products in a market where modern consumers place high value on everyday convenience.

To enhance customer loyalty and satisfaction, pet food manufacturers should improve transparency regarding product origins and manufacturing processes. As consumers show increasing concern about the safety and ethical standards of the products they purchase, providing detailed information can help build stronger trust and foster long-term relationships with customers. Transparent labeling and certifications that validate claims regarding the quality and sourcing of ingredients are essential for meeting the growing demand for ethical and responsible pet food production.

Health-related claims are another key aspect that pet food companies should emphasize in their marketing strategies. Consumers are increasingly focusing on the health and well-being of their pets, and pet food products that promote health benefits will continue to see growing demand. Pet food manufacturers should highlight the nutritional value and health benefits of their products to ensure that consumers are well-informed about the positive impacts on their pets’ health. This is particularly important for premium products that are often marketed as more nutritious and beneficial to pets.

By focusing on transparency, convenience, and health-related claims, pet food manufacturers can better align their products with consumer expectations. Doing so will help to increase customer satisfaction and loyalty, positioning companies to thrive in South Korea’s highly competitive and rapidly growing pet food market.

## 6 Conclusion

### 6.1 Academic implications

The results of this study underscore the significant impact of reliability and convenience on customer satisfaction and loyalty in the pet food market, emphasizing their critical role in shaping consumer behavior.

This study contributes to the consumer research literature by empirically validating the distinct roles of reliability and convenience among pet food selection attributes, thereby extending satisfaction–loyalty models to an underexplored product domain. The findings support the theoretical relevance of these attributes beyond traditional food categories and provide a foundation for future cross-cultural or longitudinal investigations.

This study enriches the literature by elucidating these relationships, although it is not without limitations. Notably, the demographic representation in the survey was skewed, with only 7.4% of participants aged over 50 years. This limitation is significant in the context of South Korea’s aging population, suggesting that the findings may not comprehensively reflect the preferences and behaviors of older consumers. Therefore, future research should include a more demographically representative sample to ensure generalizability across age groups.

Moreover, this study focused on a limited number of factors influencing pet food selection, satisfaction, and loyalty. Future research should expand the scope to include a wider array of attributes and enhance measurement tools to capture more detailed aspects of consumer behavior. In addition, a more granular consumer market segmentation could enable pet food companies to tailor their marketing strategies more effectively and meet diverse consumer needs more precisely.

A comparative analysis of the effects of reliability and convenience on consumer purchasing behavior in both the global and South Korean markets is also recommended. Such studies could provide deeper insights into these markets and aid South Korean pet food manufacturers in enhancing their international competitiveness. By understanding the specific demands and preferences of the different markets, South Korean brands can better position themselves globally, leverage their unique strengths, and adapt to regional consumer expectations. This strategic approach will be crucial for maintaining relevance and driving sustainable growth in the increasingly competitive global pet food industry.

### 6.2 Practical implications

From a practical standpoint, the results suggest that marketers should focus on enhancing packaging usability, storage convenience, and brand credibility, rather than relying solely on product quality or pricing strategies. Strategic efforts that emphasize transparency, trustworthiness, and consumer-centered convenience are more likely to foster strong loyalty in the increasingly premiumized pet food market.

### 6.3 Policy recommendations

At the policy level, governmental agencies and pet food industry associations in South Korea are encouraged to strengthen systems for labeling transparency, ingredient traceability, and food safety certification. This includes introducing or enhancing certified quality labeling schemes that help consumers clearly identify verified and nutritionally balanced products. Regulatory bodies should ensure that such labeling systems are standardized, easily interpretable, and enforced with oversight in view of pet owners’ increasing concerns about ingredient sourcing and safety.

It is recommended that public–private campaigns be implemented to promote awareness of brands that use high-quality, origin-verified ingredients. These campaigns should shift the consumer focus from general national origin to ingredient credibility and processing transparency, which are increasingly valued in global markets. By reinforcing consumer trust through reliable certification frameworks and transparent communication, Korean pet food products can secure a stronger position not only domestically but also internationally, contributing to the sustainable development of the industry.

## Data Availability

The raw data supporting the conclusions of this article will be made available by the authors, without undue reservation.
